# Membrane Emulsification Preparation of ADN/PVA Composite Particles Exhibiting Excellent Thermal Properties and Anti-Hygroscopicity

**DOI:** 10.3390/molecules31173136

**Published:** 2026-09-07

**Authors:** Shimin Zhang, Baoyun Ye, Xiaoying Cheng, Hongxia Zhang, Jingyu Wang

**Affiliations:** School of Environment and Safety Engineering, North University of China, Taiyuan 030051, China

**Keywords:** ammonium dinitramide, membrane emulsification, energetic composite microspheres, anti-hygroscopicity

## Abstract

Ammonium dinitramide (ADN) is a high-energy green oxidizer with significant potential for use in solid propellants; however, its practical application is restricted by its strong hygroscopicity and low safety. In this study, morphology control and surface coating were simultaneously addressed by preparing ADN/PVA composite particles with different PVA contents by membrane emulsification. At a PVA content of 5%, the ADN/PVA composite microspheres exhibited favorable morphology and a high degree of sphericity, with no phase transition induced and the crystal structure well preserved. DSC results showed that the initial decomposition temperature of ADN/PVA increased by 21.88–26.50 °C compared to that of raw ADN, and the exothermic peak became narrower, indicating that the energy release of ADN was more concentrated and its thermal stability was significantly improved. Meanwhile, the impact sensitivity and friction sensitivity were reduced by 33.33% and 16.67%, respectively, relative to raw ADN, and the moisture absorption rate was reduced by 87.92%. Therefore, the ADN/PVA composite particles prepared by membrane emulsification exhibit excellent thermal stability and anti-hygroscopicity.

## 1. Introduction

Ammonium dinitramide (ADN), a novel high-energy oxidizer [1,2], is considered an ideal candidate to replace conventional oxidizers such as ammonium perchlorate (AP) and ammonium nitrate (AN) in solid propellants and high explosives, owing to its high energy density, halogen-free composition, and clean combustion products [3]. It thus shows broad application prospects [4,5]. However, ADN exhibits extremely strong hygroscopicity [6,7], readily absorbing moisture from the ambient environment, which causes surface dissolution and performance degradation, severely impairing its storage stability [8,9]. Moreover, ADN crystals typically exhibit irregular blocky or acicular morphologies with numerous corners and defects on the particle surface, resulting in high sensitivity to mechanical stimuli [10]. Therefore, simultaneously improving the safety and anti-hygroscopicity of ADN has become a central issue in current ADN application research [11,12,13].

To address the above issues, researchers have attempted various modification strategies, mainly including spherical crystallization [14,15], surface coating [16,17,18], and co-crystallization [19,20,21]. Li et al. [22] prepared the energetic microspheres ADN@POSS-NH_2_ by a facile solvent evaporation method. The results showed that hydrophobicity was improved and hygroscopicity was remarkably reduced. In addition, POSS-NH_2_ had a catalytic effect on the thermal decomposition of ADN while having little effect on the friction sensitivity. Leng et al. [23] prepared ADN spherical particles with high sphericity, good powder properties, and an adjustable particle size distribution using quasi-emulsion solvent diffusion spherical crystallization technology. The spherical products exhibited a 47.6% lower saturated moisture absorption ratio at 25 °C with a relative humidity of 53% and a 96.1% lower caking strength after one week of storage under low-humidity conditions compared to raw ADN, which confirmed their improved anti-hygroscopic performance and anti-caking ability.

As a novel technology developed and applied in recent years, membrane emulsification has shown unique advantages in the preparation of microspheres, microcapsules, and composite particles [24,25,26]. This technique involves extruding the dispersed phase and the continuous phase through a microporous membrane, utilizing the confinement effect of the membrane pores and shear forces to produce emulsion droplets with uniform particle size and regular morphology, which are then solidified to obtain composite microspheres [27,28]. Compared with traditional mechanical emulsification methods, membrane emulsification offers advantages such as uniform droplet size, mild operating conditions, and good reproducibility. Polyvinyl alcohol (PVA) is a colloidal organic compound that readily forms films, and the resulting films exhibit excellent mechanical and chemical properties [29].

On this basis, in this study, membrane emulsification was employed to simultaneously achieve morphology control and surface coating. Through droplet confinement and interfacial assembly effects, ADN/PVA composite particles were prepared, achieving the spheroidization modification of ADN crystals and a uniform PVA coating on the particle surfaces. The effects on morphology, crystal structure, thermal decomposition characteristics, anti-hygroscopicity, and mechanical sensitivity were investigated. This work provides a feasible technical approach and reference for the high-performance modification of ADN.

## 2. Results and Discussion

### 2.1. Crystal Morphology of Microspheres

**Figure 1:**
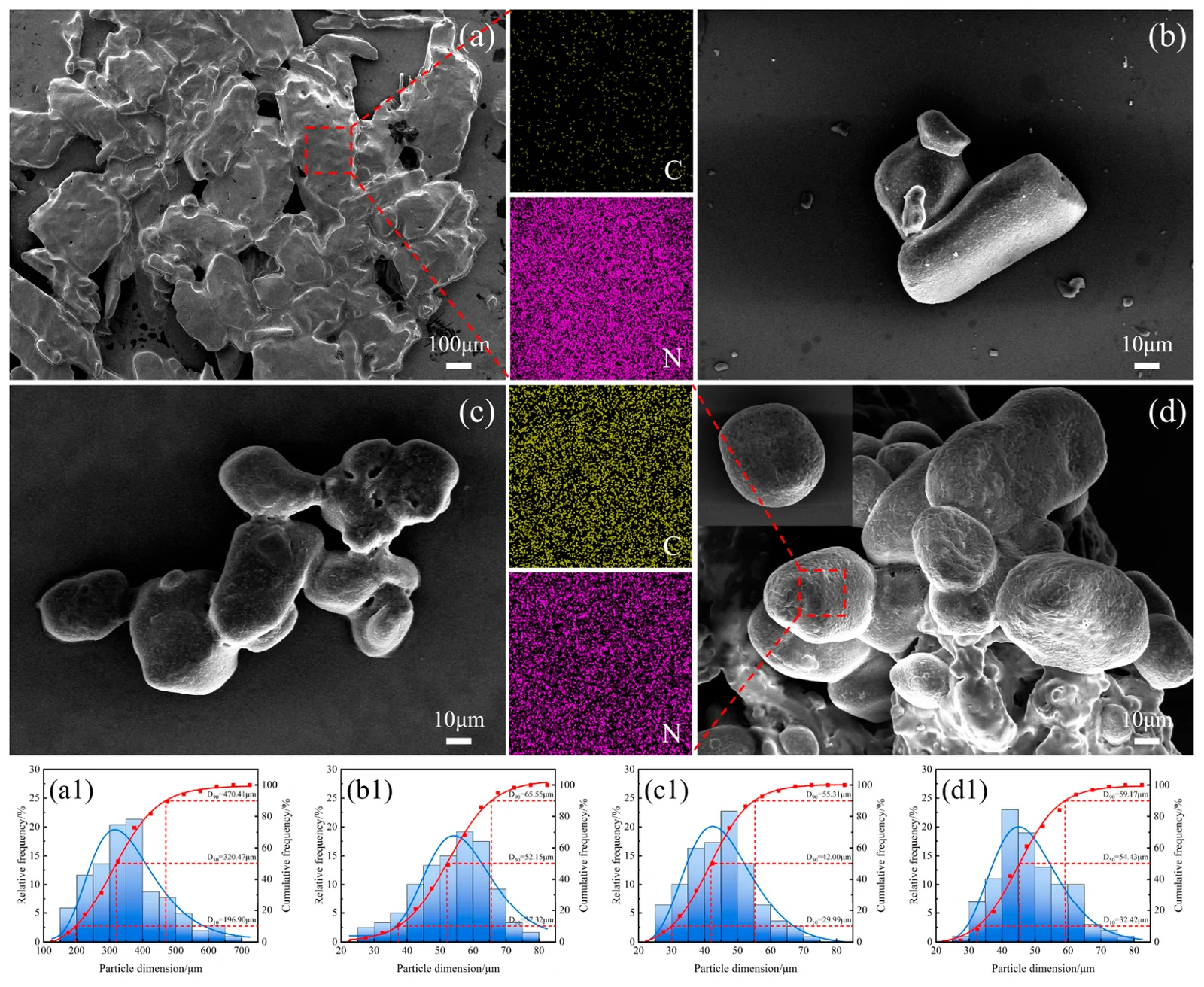
SEM images and particle size distribution of (**a**) raw ADN, (**b**) ADN/PVA (1%), (**c**) ADN/PVA (3%) and (**d**) ADN/PVA (5%), and particle size distribution of (**a1**) raw ADN, (**b1**) ADN/PVA (1%), (**c1**) ADN/PVA (3%) and (**d1**) ADN/PVA (5%).

SEM was employed to observe the micromorphology of raw ADN and ADN/PVA composite particles prepared with different PVA contents, and the SEM results are shown in Figure 1. As can be seen from the figure, the raw ADN particles exhibit a distinctly irregular blocky structure, with blurred edges and unclear contours. This phenomenon can be attributed to the strong hygroscopicity of ADN itself, which inevitably contacts air during sample preparation and testing. Water molecules in the air are readily adsorbed onto the surface of ADN particles, rapidly triggering the hydrolysis reaction of ADN. The hydrolysis process preferentially occurs at surface defects and corners, causing dissolution of the superficial structure, which in turn blurs the crystal edges of raw ADN and impairs its morphological integrity.

In contrast, the ADN/PVA composite particles prepared by membrane emulsification exhibit clear edges and show no signs of hydrolysis, indicating that the PVA coating layer can effectively block direct contact between ADN and moisture in the air, thereby inhibiting the hydrolysis reaction. Further observation of the effect of different PVA contents on the morphology of the ADN/PVA composite particles reveals that as the PVA mass fraction increases from 1% to 5%, the particle morphology gradually becomes more regular. This is attributed to the good film-forming property of PVA, which uniformly coats the surface of ADN crystals during membrane emulsification, forming a continuous and dense polymer film, with the C element being homogeneously distributed. This film not only serves as a physical barrier but also regulates the growth morphology of ADN crystals through interfacial effects. During crystallization, PVA molecular chains adsorb onto the crystal faces of ADN, thereby reducing the surface energy and promoting isotropic crystal growth. When the PVA content reaches 5%, the morphology of ADN/PVA tends toward spheroidization; the median particle size D_50_ is 54.43 μm, and at this concentration, the coating and regulating effects of PVA are optimal.

### 2.2. Moisture Absorption Test

**Figure 2:**
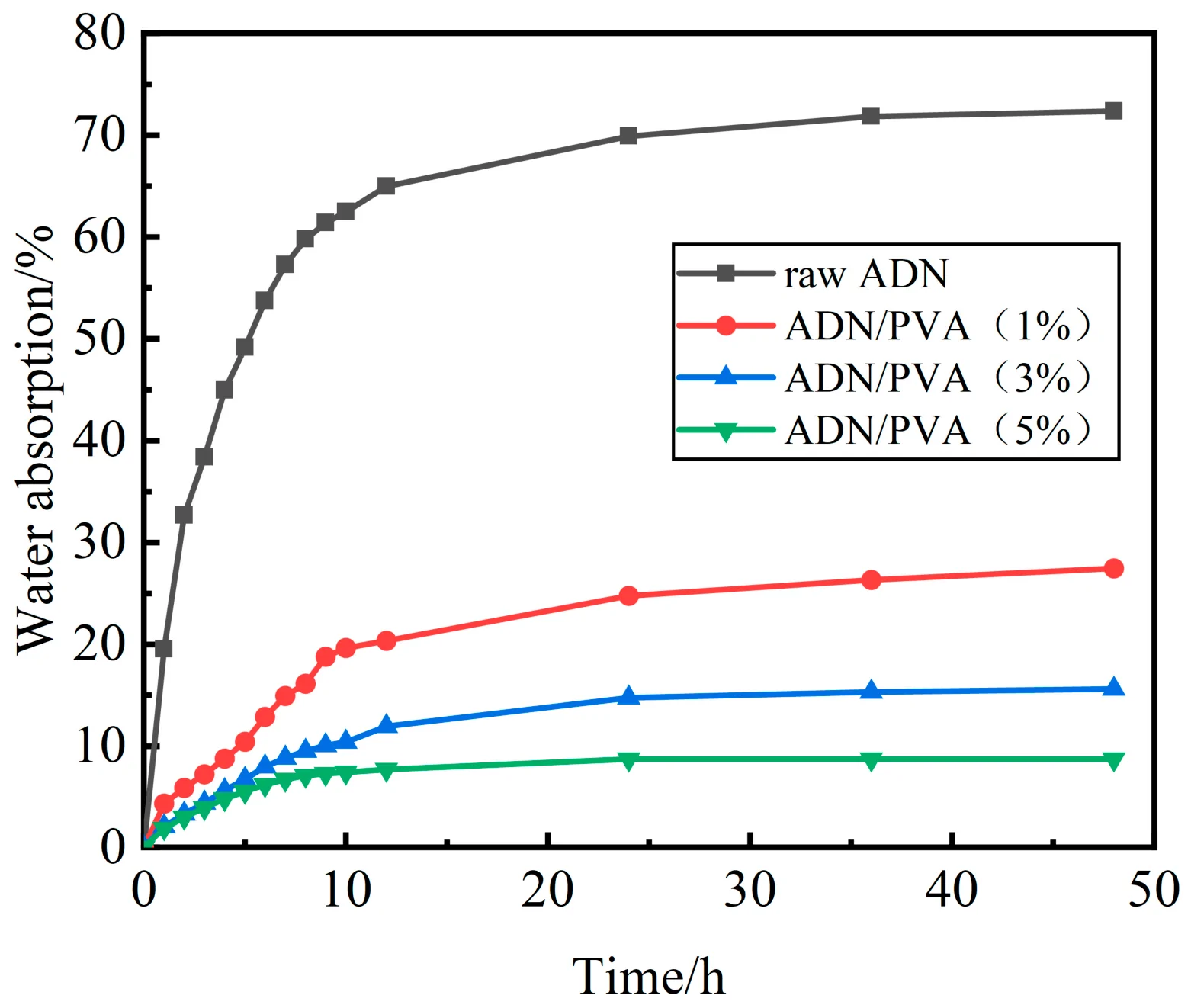
Moisture absorption curves of raw ADN and ADN/PVA.

The moisture absorption curves were measured under environmental conditions of 70% relative humidity and 25 °C for 48 h, and the test results are shown in Figure 2.

Raw ADN exhibits strong hygroscopicity. At the initial stage of the test, the moisture absorption rate increases rapidly, presenting a fast moisture absorption phase. Subsequently, the moisture absorption rate gradually slows down but still maintains a continuous increasing trend, reaching 72.34% at 48 h. In contrast, the hygroscopicity of the ADN/PVA composite is significantly reduced. Throughout the 48 h testing period, its moisture absorption rate remains at a relatively low level, with values of 27.47%, 15.63%, and 8.74% for ADN/PVA (1%), ADN/PVA (3%), and ADN/PVA (5%), respectively, which are far lower than that of raw ADN. Moreover, the moisture uptake decreased with increasing PVA content. Notably, for ADN/PVA (5%), the moisture gain leveled off after 8 h of absorption, and the slope of the gain curve continued to decrease, indicating that water diffusion in the composite had approached a steady state and that the PVA layer did not undergo obvious failure due to osmotic accumulation. Thus, the composite maintained its low hygroscopicity. This result indicates that the introduction of PVA significantly improves the anti-hygroscopicity of ADN. This is because PVA, as a high-molecular-weight polymer, forms a continuous and dense polymer coating layer on the surface of ADN crystals. This physical barrier effectively isolates ADN from direct contact with water molecules, thereby reducing the moisture absorption rate. Meanwhile, PVA modification causes the crystal morphology of ADN to tend toward regular sphericity, with smooth particle surfaces and significantly reduced crystal defects and edges, resulting in a denser structure that hinders the diffusion channels of water molecules into the material, thereby effectively suppressing the moisture absorption process. This excellent anti-hygroscopic performance is of great significance for improving the environmental stability and storage safety of ADN in practical applications. Therefore, in combination with the SEM results, the ADN/PVA (5%) sample was selected for further performance characterization.

### 2.3. Crystalline Form Analysis

**Figure 3:**
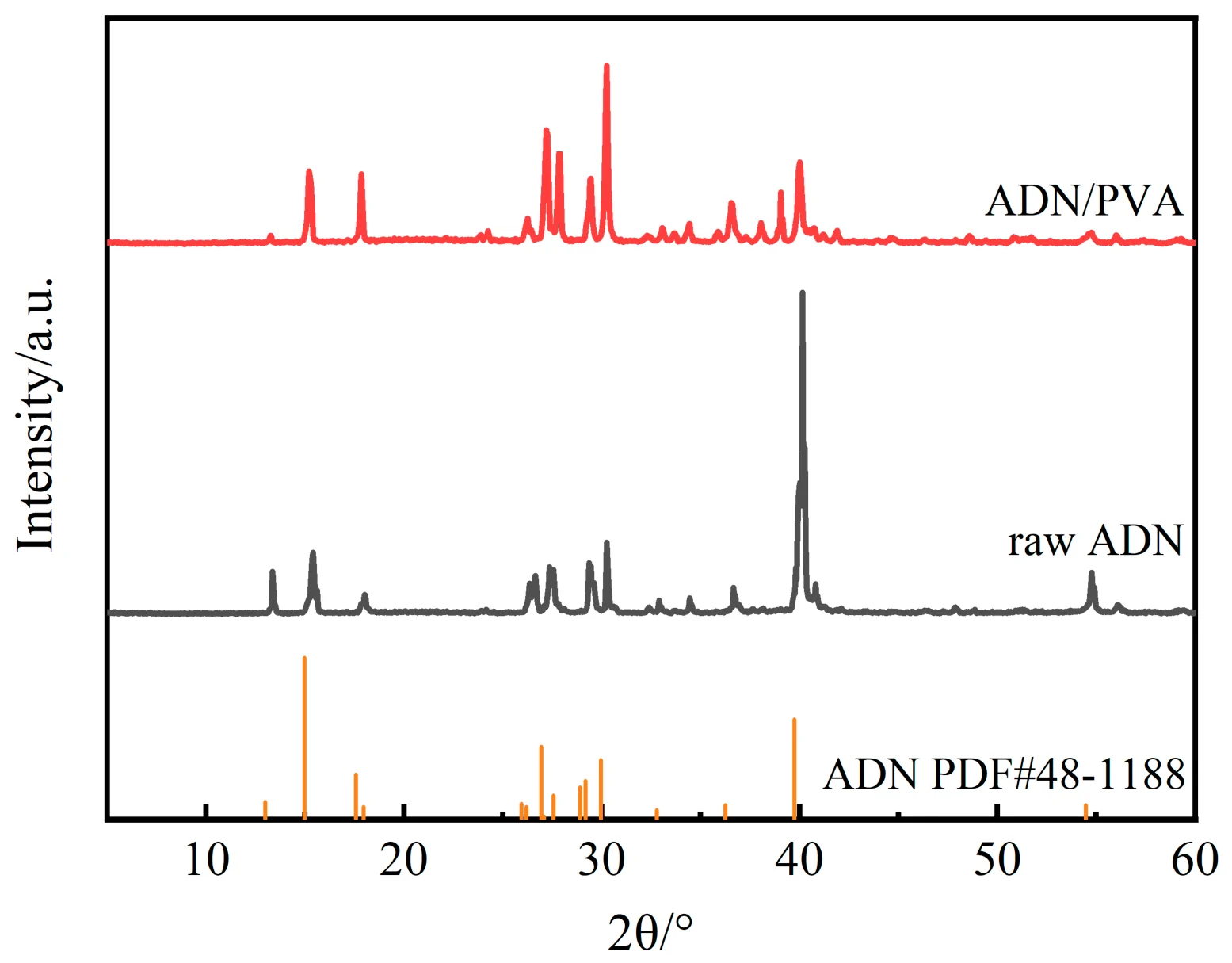
XRD patterns of raw ADN and ADN/PVA (5%).

In order to explore the effect of PVA coating and membrane emulsification on the crystal structure of ADN, the prepared samples were analyzed by X-ray powder diffraction (XRD), and the diffraction patterns were compared with the standard PDF card of ADN (PDF No. 48-1188) [30,31]. The results are shown in Figure 3.

As shown in Figure 2, the raw ADN sample exhibits clear characteristic diffraction peaks at 2θ = 13.3°, 15.4°, 18.0°, 26.5°, 26.7°, 27.3°, 29.4°, 30.2°, 40.1°, and 54.7°, which are in good agreement with the standard PDF card. In contrast, the diffraction pattern of the ADN/PVA composite sample completely retains all the above-mentioned characteristic peaks, with no new diffraction peaks appearing or existing peaks disappearing, and no obvious shift in peak positions is observed. These results fully demonstrate that during the membrane emulsification process, the introduction of PVA and its film-forming coating did not damage the crystal structure of ADN or induce polymorphic transformation, amorphization, or other phase transition behaviors, and the crystal structure of ADN was well preserved in the composite.

Although the diffraction peak positions are generally consistent, the relative intensities of the diffraction peaks for the ADN/PVA sample are significantly different from those of raw ADN. For raw ADN, the diffraction peak near 2θ = 40.1° exhibits the highest intensity among all peaks, serving as the strongest characteristic peak. In contrast, for the ADN/PVA composite sample, the intensity differences among the diffraction peaks are markedly reduced. This change in relative intensities does not originate from alterations in the crystal structure. Specifically, the intensity of XRD peaks depends not only on the crystal structure factor but is also strongly governed by the orientation arrangement of crystal planes within the measurement plane. Raw ADN crystals exhibit irregular blocky or acicular morphologies, and both blocky and acicular crystals tend to lie flat naturally on the XRD sample stage, giving rise to a strong preferred orientation. In contrast, spherical particles are randomly oriented in space during packing, which eliminates preferred orientation. Therefore, the spheroidization of the ADN/PVA composite samples corrects the severe preferred orientation present in the scans of the raw needle-like ADN.

### 2.4. Chemical Structure Analysis

**Figure 4:**
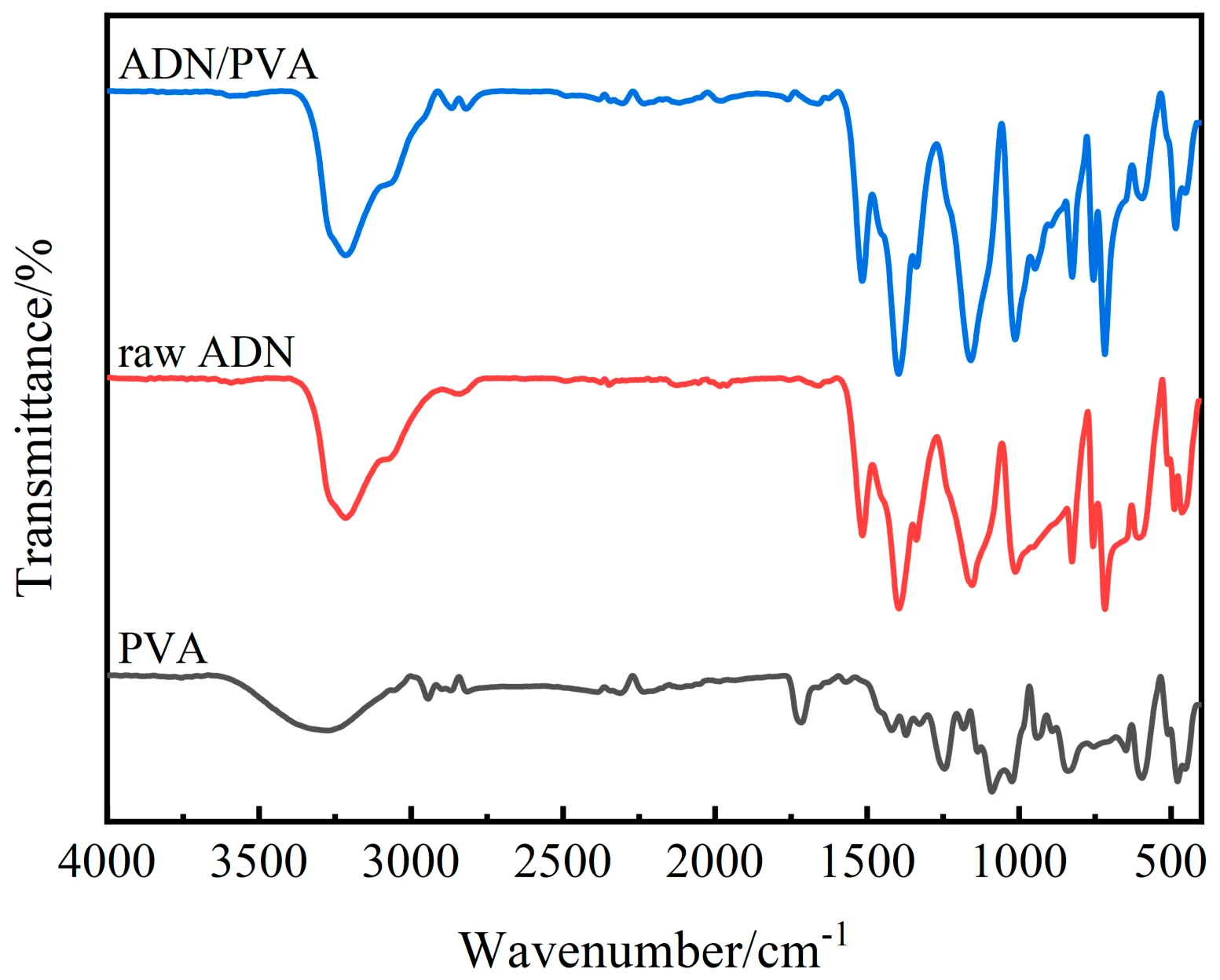
FTIR spectra of raw ADN and ADN/PVA (5%).

Figure 4 shows the FTIR spectra of different samples. For raw ADN, the absorption peak at 3214 cm^−1^ is attributed to the vibrational absorption of NH_4_^+^, corresponding to the N-H stretching vibration in the ammonium ion. The peaks at 1514 cm^−1^ and 1338 cm^−1^ are assigned to the vibrational absorptions of nitro groups, corresponding to the asymmetric and symmetric stretching vibrations of –NO_2_, respectively. The peak at 1011 cm^−1^ arises from the characteristic absorption of the N-N-N stretching vibration [32]. In the FTIR spectrum of PVA, a broad and strong band appears in the range of 3500–3600 cm^−1^, which corresponds to the O-H stretching vibration. The peaks at 2944 cm^−1^ and 2898 cm^−1^ are attributed to the asymmetric and symmetric stretching vibrations of CH_2_, respectively. The peak at 1717 cm^−1^ is assigned to the C=O stretching vibration, and the peak at 1090 cm^−1^ corresponds to the C-O stretching vibration [33].

The spectrum of the ADN/PVA composite microspheres exhibits the characteristic peaks of ADN as the dominant features, superimposed with those of PVA, confirming the coexistence of both components in the composite. Compared with raw ADN, all characteristic peak positions, shapes, and relative intensities of ADN in the ADN/PVA composite remain essentially unchanged, with neither new peaks appearing nor original peaks disappearing, indicating that the interaction between PVA and ADN is merely physical blending, and PVA does not alter the chemical structure of ADN.

### 2.5. Thermal Decomposition Performance

**Figure 5:**
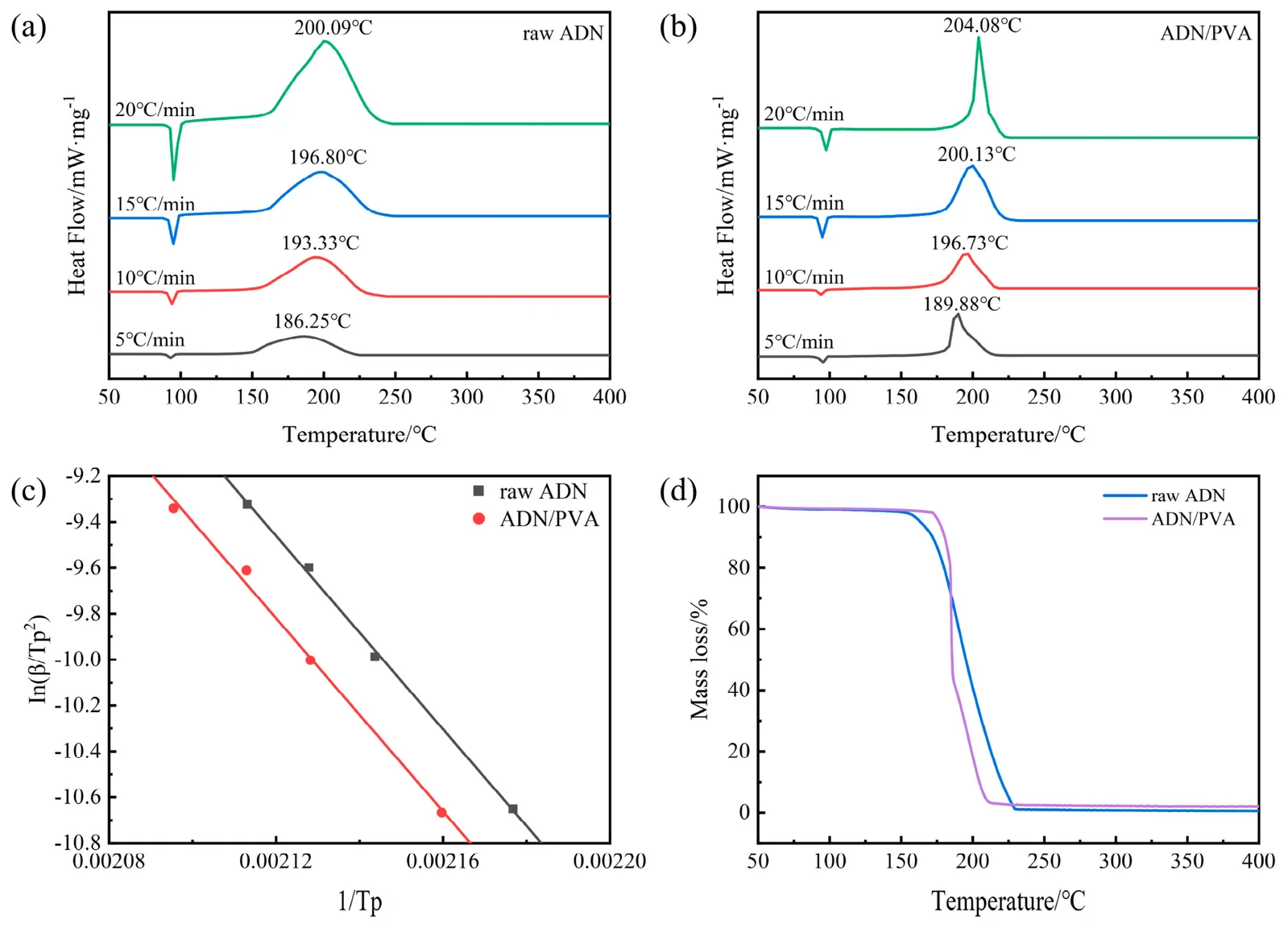
DSC curves of (**a**) raw ADN and (**b**) ADN/PVA (5%), (**c**) fitted curve, and (**d**) TG curve.

TG-DSC is an important characterization technique for studying the thermal decomposition performance and decomposition reaction kinetics of energetic materials. The exothermic curves of the raw ADN and the ADN/PVA composite were measured by DSC at four different heating rates, 5 °C·min^−1^, 10 °C·min^−1^, 15 °C·min^−1^, and 20 °C·min^−1^, as shown in Figure 5.

Both raw ADN and the ADN/PVA composite exhibit a typical two-stage thermal behavior. An endothermic peak appears at around 100 °C, which corresponds to the melting peak as the material transforms from the crystalline solid state to the liquid phase. As the temperature gradually increases, a strong exothermic peak appears at around 200 °C, indicating the complete decomposition of ADN into NO_2_, NO, ammonia, water, and other products [34]. Moreover, as the heating rate increases from 5 °C·min^−1^ to 20 °C·min^−1^, the decomposition peak temperatures show a systematic and monotonic increase. The peak temperature of raw ADN shifts from 186.25 °C to 200.09 °C, while that of ADN/PVA shifts from 189.88 °C to 204.08 °C. This behavior is consistent with the general characteristics of non-isothermal thermal analysis: the higher the heating rate, the more significant the thermal hysteresis effect, the shorter the time required for the sample to reach thermal equilibrium, and the greater the shift of the decomposition reaction toward higher temperatures.

The onset temperature, peak temperature, and end temperature of the exothermic peaks for raw ADN and the ADN/PVA composite are listed in Table 1. It can be seen that the exothermic interval of ADN/PVA is narrower than that of raw ADN. The initial decomposition temperature of ADN/PVA increased by 21.88–26.50 °C compared with that of raw ADN. This observation indicates that the PVA coating layer can effectively block the direct transfer of heat to the ADN crystals, delay the initial decomposition reaction, and thus improve the thermal stability of ADN. However, despite the significant increase in onset temperature, the peak temperature of ADN/PVA is only 3.33–3.99 °C higher than that of raw ADN. Meanwhile, the end temperature is markedly reduced, and the thermal decomposition temperature range is substantially narrowed.

This phenomenon indicates that PVA exerts a certain regulatory influence on the thermal decomposition process of ADN. The physical barrier effect of the PVA shell delays heat transfer to ADN, thereby raising the initial decomposition temperature. Once sufficient heat has accumulated to overcome the onset reaction energy barrier of ADN, the decomposition reaction can proceed rapidly to completion, resulting in a significantly narrowed decomposition temperature range. Therefore, the PVA coating modification effectively enhances the thermal stability of ADN, and the marked increase in the onset decomposition temperature has positive implications for safe handling in practical applications.

As shown in the TG curves (Figure 5d), both raw ADN and ADN/PVA exhibit a one-step thermal decomposition reaction, with no mass loss occurring during the endothermic melting stage around 100 °C. The T_5%_ (temperature at 5% mass loss) of raw ADN is approximately 163 °C, while that of ADN/PVA is about 176 °C, representing an increase of roughly 13 °C. This indicates that the PVA coating layer exerts a protective effect on the ADN particles, delaying the initiation of thermal decomposition—that is, the coating improves the thermal stability of ADN. Moreover, the mass loss curve of ADN/PVA is steeper than that of raw ADN, which is consistent with the DSC observations. Once decomposition is initiated, the reaction proceeds more intensively and rapidly. Meanwhile, raw ADN decomposes completely, leaving virtually no residual mass, while ADN/PVA retains approximately 2% residue, which is attributed to the carbonization of PVA and indirectly confirms the presence of the coating layer.

To further investigate the detailed parameters of thermal decomposition, the apparent activation energies (*Ea*) of raw ADN and the as-prepared ADN composites were calculated using the Kissinger Equation (1) [35], and the calculated results are listed in Table 2. The plots of ln(*β/T_p_*^2^) versus *1/T_p_* are shown in Figure 5c.

(1) $$\ln\frac{\beta_{i}}{T_{\mathrm{pi}}^{2}}=\ln\frac{\mathrm{AR}}{E_{a}}-\frac{E_{a}}{{\mathrm{RT}}_{\mathrm{pi}}}$$

where *β_i_* is the heating rate in °C·min^−1^; *Ea* is the apparent activation energy in kJ·mol^−1^; *A* is the pre-exponential factor; *R* is the universal gas constant, *R* = 8.314 J·mol^−1^·K^−1^; and *T_pi_* is the exothermic peak temperature in K obtained from the DSC curves.

The thermal explosion critical temperature reflects the thermal stability of energetic materials. The thermal stability parameters were calculated using Equations (2) and (3). The thermodynamic parameters were calculated using Equations (4)–(6) [36], and the calculated results are listed in Table 2.

(2) $$T_{p0}=T_{\mathrm{pi}}-{b\beta }_{i}-{c\beta }_{i}^{2}$$

(3) $$T_{b}=\frac{E_{a}-\sqrt{E_{a}^{2}-4RE_{a}T_{p0}}}{2R}$$

(4) $$\Delta H^{\neq }=E_{a}-RT_{p0}$$

(5) $$\mathrm{Aexp}\frac{-E_{a}}{RT_{p0}}=\frac{kT_{p0}}{h}\exp\frac{-\Delta G^{\neq }}{RT_{p0}}$$

(6) $$\Delta S^{\neq }=\frac{\Delta H^{\neq }-\Delta G^{\neq }}{T_{p0}}$$

where *T_p_*_0_ is the decomposition peak temperature when the heating rate approaches zero in K; *T_b_* is the thermal explosion critical temperature in K; *b* and *c* are undetermined coefficients; Δ*H*^≠^ is the activation enthalpy in kJ·mol^−1^; Δ*G*^≠^ is the activation free energy in kJ·mol^−1^; Δ*S*^≠^ is the activation entropy in J·mol^−1^·K^−1^; *k* is the Boltzmann constant, *k* = 1.381 × 10^−23^ J·K^−1^; and *h* is the Planck constant, *h* = 6.626 × 10^−34^ J·s^−1^.

As shown in Table 2, the *Ea* values of raw ADN and ADN/PVA are 175.23 kJ·mol^−1^ and 175.05 kJ·mol^−1^, respectively, which are almost equal. This indicates that the introduction of PVA does not alter the intrinsic activation energy barrier corresponding to the decomposition reaction of ADN. Meanwhile, the *T_p_*_0_ and *T_b_* of ADN/PVA increased by 3.45 K and 3.61 K, respectively, compared with those of raw ADN, demonstrating that the PVA coating layer indeed provides effective thermal protection for ADN under dynamic heating conditions, shifting the thermal decomposition behavior toward a higher temperature range.

As shown in Table 2, the activation enthalpies (Δ*H*^≠^) of raw ADN and ADN/PVA are essentially identical, since Δ*H*^≠^ is determined by *Ea* within a similar temperature range. The Δ*S*^≠^ of ADN/PVA is 3.45 J·mol^−1^·K^−1^ lower than that of raw ADN. According to transition state theory, the activation entropy reflects the degree of disorder of the system as reactant molecules transition from the ground state to the transition state. A positive Δ*S*^≠^ indicates that the decomposition of ADN is a process in which the molecular degrees of freedom increase and the system tends toward disorder, whereas the decrease in Δ*S*^≠^ for ADN/PVA suggests that the spatial confinement provided by the PVA coating partially restricts the freedom of molecular motion when ADN molecules transition from the ground state to the transition state, rendering the system more ordered. Meanwhile, Δ*G*^≠^ serves as a comprehensive indicator for judging the spontaneity of a reaction; the higher its value, the more difficult it is for the reaction to proceed. The Δ*G*^≠^ of ADN/PVA is slightly higher than that of raw ADN. Although the increase is small, this trend is consistent with the increases in *T_p_*_0_ and *T_b_*, indicating from a thermodynamic perspective that the PVA coating imposes a higher free energy barrier on the ADN decomposition reaction, thereby enhancing its thermal stability.

### 2.6. Mechanical Sensitivity

**Figure 6:**
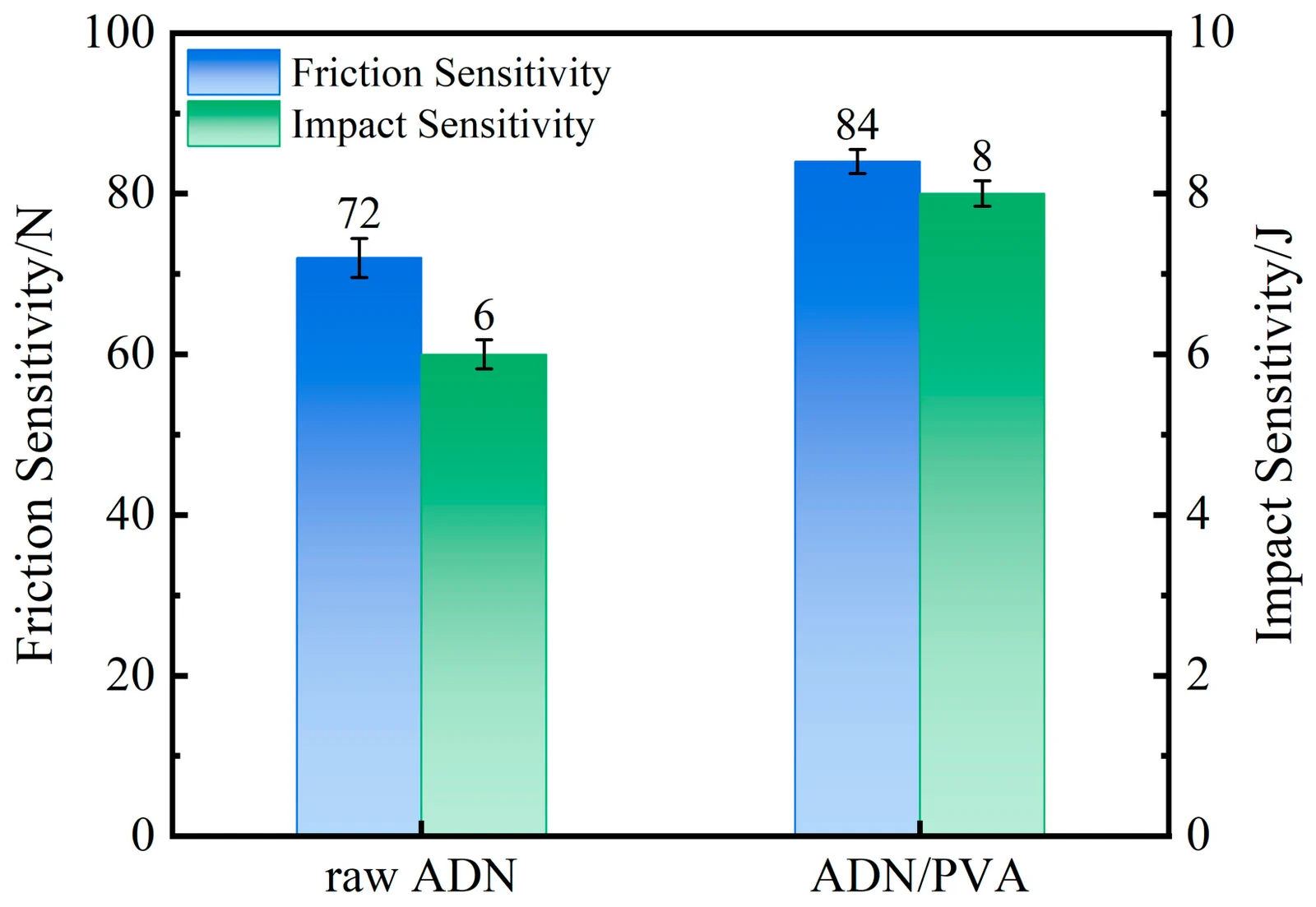
Friction and impact sensitivities of raw ADN and ADN/PVA.

Mechanical sensitivity, particularly the response characteristics to external impact and friction stimuli, is one of the core indicators for evaluating the safety performance of energetic materials. In this study, the critical impact energy and critical friction energy of raw ADN and the as-prepared ADN/PVA particles were tested, and the test results are shown in Figure 6.

The test results show that the impact sensitivities of raw ADN and ADN/PVA are 6 J and 8 J, respectively, and the friction sensitivities are 72 N and 84 N, respectively. The PVA coating modification effectively reduced the mechanical sensitivity of ADN and improved its safety. This is because the PVA coating layer forms a continuous and dense film on the surface of ADN crystals, reducing the exposed active sites on the crystal surface. When the composite particles are subjected to external impact loading, the polymer film layer acts as a buffer medium that can absorb and dissipate part of the impact energy, delay its propagation within the crystal, and effectively hinder the formation of hot spots. Meanwhile, the ADN/PVA particles exhibit a high degree of sphericity. When subjected to external friction loading, the effective friction contact area is greatly reduced, and the particles tend to undergo relative sliding under stress, thereby reducing frictional heat generation and stress concentration. In summary, PVA can form a protective film on the ADN surface, providing excellent protection against external mechanical stimuli and making ADN more insensitive.

### 2.7. Theoretical Calculation

In practical solid propellant formulations, ADN serves as a high-energy oxidizer, and its value lies in enhancing the specific impulse of the propellant. A scientific evaluation of energetic composite materials must strike a balance between safety and practical applicability. Raw ADN and ADN/PVA were incorporated into a commonly used PBT-based solid propellant formulation, and the resulting compositions were designated as CSP-1 and CSP-2, respectively. The specific contents of the formulations are listed in Table 3.

Theoretical parameters of different samples in composite solid propellants were calculated using EXPLO5 software, including specific impulse (*I_sp_*), oxygen balance (*OB*), enthalpy of formation (∆*H*), isobaric combustion temperature (*T_c_*), heat of isobaric combustion (*H_c_*), mean molecular mass of gaseous products ($\bar{M}$), and characteristic exhaust velocity (C*). The calculation results are listed in Table 4.

As shown in Table 4, compared with CSP-1, the introduction of the PVA component in CSP-2 leads to a decrease in oxygen balance from −21.09% to −26.98%, along with slight reductions in isobaric combustion temperature and isobaric combustion heat (*T_c_* decreases by approximately 3.0%, and *H_c_* by about 2.0%). However, the characteristic exhaust velocity (C*) is slightly increased. More importantly, the core energy indicator—specific impulse (*I_sp_*)—remains almost unaffected. This indicates that the ADN/PVA modification not only significantly enhances moisture resistance but also causes no substantial damage to the propellant’s intrinsic energy performance. Thus, it maintains the energy level of the propellant while effectively overcoming the hygroscopicity bottleneck in the practical application of ADN, demonstrating the feasibility of this modification strategy for real-world applications.

## 3. Materials and Methods

### 3.1. Materials

Raw ADN (99.9% purity) was purchased from Liming Chemical Research Institute. PVA1799 was provided by Shandong Qingdao Yousuo Chemical Technology Co., Ltd., Qingdao, China. Poly(ethylene glycol) dioleate (PEG400DO) and decane were supplied by Shanghai Aladdin Biochemical Technology Co., Ltd., Shanghai, China. The membrane emulsification equipment used was model FM0210/500M, manufactured by Zhongke Senhui Microsphere Technology (Suzhou) Co., Ltd., Suzhou, China.

### 3.2. Preparation of ADN/PVA Energetic Microspheres

(1) Preparation of the aqueous phase

A certain mass of ADN was dissolved in deionized water to prepare a 75 wt% ADN aqueous solution. Then, PVA with mass fractions of 1%, 3%, and 5% relative to the mass of ADN was added, respectively, to form the aqueous phase.

(2) Preparation of the oil phase

A certain mass of PEG400DO was added to decane to prepare a 1 wt% solution. The mixture was stirred with a magnetic stirrer at 40 °C and 400 rpm until the PEG400DO was completely dissolved, thus forming the oil phase.

(3) Preparation by membrane emulsification

**Figure 7:**
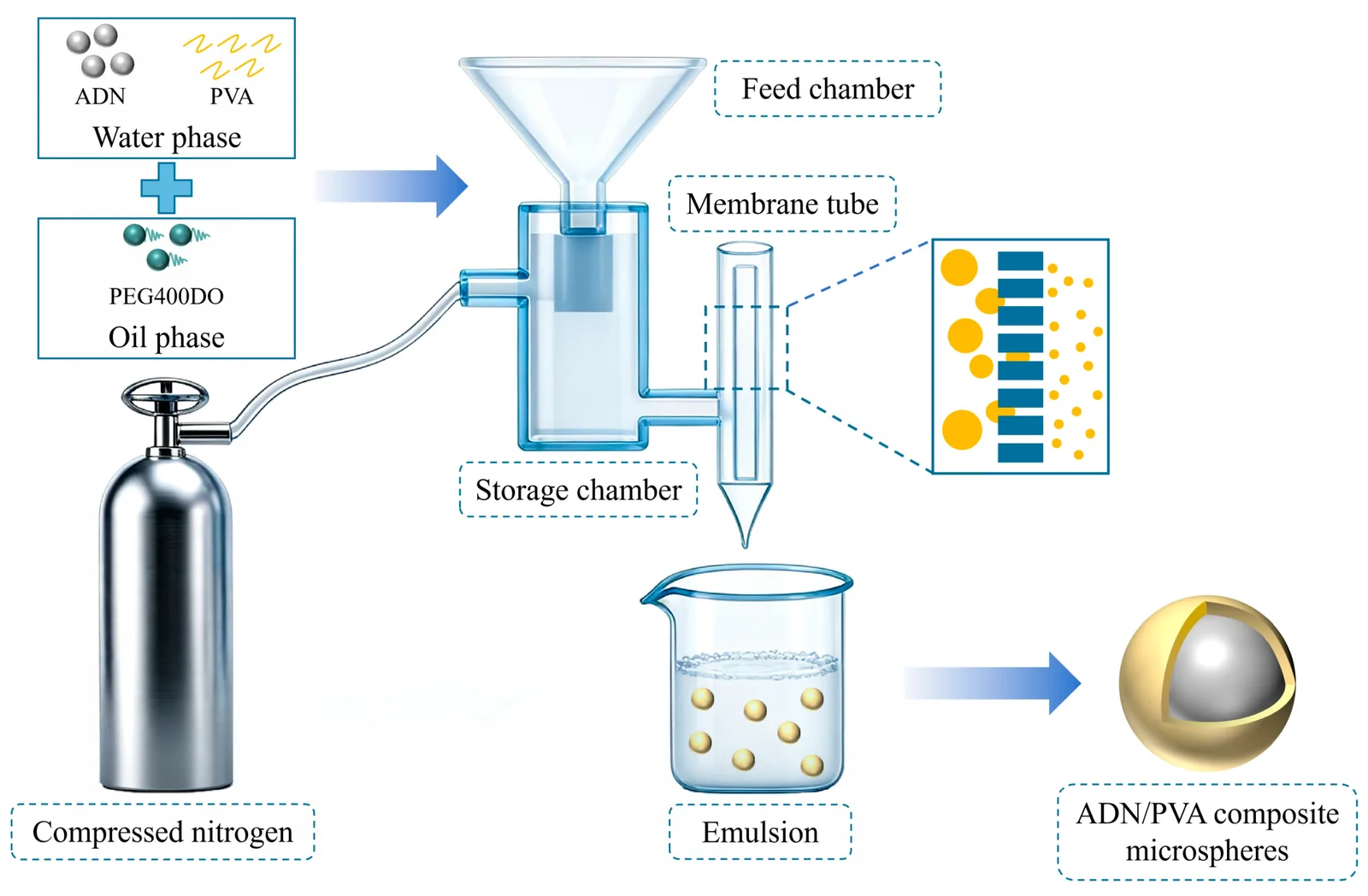
Schematic diagram of membrane emulsification.

The prepared aqueous phase was poured into the oil phase at a volume ratio of 1:4 (aqueous/oil), and the mixture was stirred to form a pre-emulsion. The obtained pre-emulsion was then placed into a membrane emulsification device, into which nitrogen was introduced, and the pressure was set at 0.02 MPa. The pre-emulsion was passed through a ceramic membrane tube with a pore size of 50 μm for three cycles, yielding a W/O emulsion with uniform droplets. The membrane emulsification process is schematically illustrated in Figure 7.

(4) Preparation of ADN/PVA composite particles

The resulting W/O emulsion was treated by a rotation-assisted solvent evaporation process at a temperature of 40 °C and a stirring rate of 75 rpm for 6 h and then filtered to collect the ADN/PVA composite particles.

### 3.3. Characterization and Measurements

The surface morphology, dimensions, and microstructure of the samples were characterized by scanning electron microscopy (SEM, MIRA3, TESCAN, Brno, Czech Republic). Samples were coated via gold sputtering prior to SEM analysis. The crystal structures were determined by powder X-ray diffraction (XRD, DX-2700, Dandong Haoyuan Corporation, Dandong, China). The analysis parameters were as follows: 2θ test angle 5–60°, voltage 40 kV, current 30 mA, and Cu-Kα radiation. The thermal decomposition curves of the samples were obtained using a differential scanning calorimeter (DSC, DSC-3, METTLER TOLEDO, Greifensee, Switzerland). DSC measurements were performed with a sample mass of 0.7 mg, an N_2_ flow rate of 50 mL·min^−1^, and heating rates of 5, 10, 15, and 20 °C·min^−1^ within the temperature range of 50 to 400 °C. The friction sensitivity and impact sensitivity of the samples were tested according to GB/T21566-2008 [37] and GB/T21567-2008 [38] using a BAM friction sensitivity meter (Addisain (Beijing) Technology Co., Ltd., Beijing, China) and a BAM impact sensitivity meter (Addisain (Beijing) Technology Co., Ltd., Beijing, China). The raw ADN and ADN/PVA were placed in a constant-humidity and constant-temperature environment (WS 70 III, Shanghai Establishment Instrument Co., Ltd., Shanghai, China) and periodically weighed to measure their hygroscopicity according to the weighing method. The hygroscopicity tests were performed in triplicate, and the average values were taken from the three parallel experiments.

## 4. Conclusions

(1) Three types of ADN/PVA composite particles with different PVA contents (1%, 3%, and 5%) were prepared by premix membrane emulsification. As the PVA mass fraction increased from 1% to 5%, the morphology of the ADN/PVA particles gradually became more regular, and the aspect ratio decreased. At a PVA content of 5%, the ADN/PVA composite microspheres exhibited favorable morphology and a high degree of sphericity.
(2) The T_5%_ of ADN/PVA (5%) increased by approximately 13 °C compared with that of raw ADN, indicating that the introduction of PVA delays the initiation of thermal decomposition and thus improves the thermal stability of ADN.
(3) The mechanical sensitivity of the prepared ADN/PVA (5%) composite was reduced. Compared with raw ADN, the critical impact energy of the composite particles increased by 2 J, and the critical friction force increased by 12 N.
(4) After being placed under environmental conditions of 70% relative humidity and 25 °C for 48 h, the moisture absorption rate of ADN/PVA (5%) decreased by 87.92% compared with that of raw ADN.

## Figures and Tables

**Table 1:** Parameters of exothermic peaks for raw ADN and ADN/PVA.

Sample	Heating Rate/°C·min^−1^	Onset Temperature/°C	Peak Temperature/°C	End Temperature/°C
Raw ADN	5	150.31	186.25	221.94
	10	156.43	193.33	229.74
	15	158.97	196.80	234.04
	20	162.18	200.09	239.55
ADN/PVA	5	176.81	189.88	215.76
	10	178.31	196.73	218.61
	15	181.23	200.13	218.70
	20	185.69	204.08	222.29

**Table 3:** Basic composition of solid composite propellants.

Component	PBT/%	TDI/%	A3/%	ADN/%	ADN/PVA/%	Al/%	Other/%
CSP-1	8.10	0.25	12.54	61.50		17.50	0.11
CSP-2	8.10	0.25	12.54		61.50	17.50	0.11

**Table 4:** Theoretical parameters of composite solid propellants.

Sample	*I_sp_*/s	*OB*/%	∆*H*/kJ·kg^−1^	*T_c_*/K	*H_c_*/kJ·kg^−1^	$\bar{M}$	C*/m·s^−1^
CSP-1	273.54	−21.09	−937.41	3723.3	−6303.36	19.24	1586.60
CSP-2	273.47	−26.98	−1044.96	3610.8	−6177.49	18.57	1587.50

**Table 2:** Thermal decomposition parameters of raw ADN and ADN/PVA.

Sample	*Ea*/kJ·mol^−1^	*T_p_*_0_/K	*T_b_*/K	Δ*H*^≠^/ kJ·mol^−1^	Δ*S*^≠^/ J·mol^−1^·K^−1^	Δ*G*^≠^/ kJ·mol^−1^
raw ADN	175.23	445.28	455.11	171.53	119.05	118.52
ADN/PVA	175.05	448.73	458.72	171.32	115.60	119.44

## Data Availability

The original contributions presented in this study are included in the article.
